# Notes and News

**Published:** 1901-06-15

**Authors:** 


### Notes and News.

HOSPITAL MEETING.

Clapham Maternity Hospital, 41 Jeffreys Road, S.W.—Annual Meeting, Wednesday, June 19th, at 3.30 p.m.

The Second Livingston Exhibition, which is practically a travellers' health exhibition, will be opened at the Westminster Town Hall on Tuesday, June 18th, and remain open until the 21st. It is held in connection with the Livingston College. A complete display of useful equipments and accessories for travel in the tropics will be on view, and interesting lectures will be given daily. Many relics and objects of interest will be shown. A medical conversazione will be held on June 20th.

The Countess Howe has received from Surgeon Lieutenant-Colonel Kilkelly, C.M.G., P.M.O. of the Imperial Yeomanry Hospital at Pretoria, a cable informing her of the sad deaths of ward orderlies T. H. Banks and J. H. Sewell, who died from enteric contracted while nursing enteric patients in the Pretoria Hospital. Dr. Richmond, of Guy's Hospital, sailed on the 4th inst. to take up his duties as principal medical officer of the new branch opened by the Imperial Yeomanry Hospitals Committee at Elandsfontein.

The principal arrangements for the forthcoming British Congress on Tuberculosis, commencing July 22nd, are now nearly completed. The programme is well thought out and very comprehensive. The subjects are divided into four sections. The first deals with the part played by State and municipality in safeguarding the public. The second embraces medical aspects, climate in relation to consumption, and sanatoria for its treatment. The third section is devoted to the pathological and bacteriological side of the questions, and the fourth includes the whole subject of tuberculosis in animals. Public addresses will be given by Professors Koch of Berlin, Brouardel of Paris, and McFadyean of the Royal Veterinary College. A valuable museum will assist members of the Congress, and social entertainments are also to be provided. The fee for membership is £1, and the offices of the committee are at 20 Hanover Square.

The West London Medico-Chirurgical Society will hold a conversazione at the Town Hall, Hammersmith, on Friday, June 21st, when Sir Richard Douglas Powell will deliver a lecture on "Acute Cardiac Failure."

The new Pathological Institute at the London Hospital will be formally opened by Sir Henry Roscoe on Wednesday, July 10th, at three o'clock. This ceremony will be followed by the Distribution of Prizes in the Library of the College.

A new isolation hospital has just been opened for Malvern, about three miles from the town, at Halfkey. The accommodation is for 22 patients. The cost is estimated at upwards of £10,000, which brings the expenditure to upwards of £500 per bed.

On Thursday, the 20th instant, at 4 p.m., the new operating theatres and children's ward at St. Thomas's Hospital are to be opened by Lord Lister; and on the same afternoon at 5 p.m. the new wing at the Poplar Hospital for Accidents will be formally opened.

At St. Andrew's Church, Holborn Circus, on Wednesday, June 19th, at 4 o'clock, a memorial tablet will be unveiled to the late Dr. William Marsden, who founded the Royal Free and the Cancer Hospitals. The tablet, which will be placed in St. Andrew's by the Company of Cordwainers, bears a record of his career and an appreciation of his character. Dr. Marsden died in 1867.

The late Lord Wantage, whose death has just been recorded, took a very active part in charitable organisations for the relief of the sick. He was chairman of the Charing Cross Hospital, and, when Sir Robert Loyd-Lindsay, took an active part in the organisation of Red Cross hospitals, and worked on British Red Cross committees during both British and foreign campaigns.

The report of the Medical Sickness and Accident Society states that during the year 1900 the number of members increased to 2,006, and the financial results of the year's working were very satisfactory. The Society is strictly confined to registered members of the medical profession, and is conducted on purely mutual principles. In 1900, £8,250 was disbursed to the members as sickness benefit.

Major-General Eaton points out that practical assistance can be given to the men now returning from South Africa by the gift of discarded clothes. In almost every case in which a man applies to the Soldiers and Sailors Help Society for aid to get employment, the applicant invariably points out that his great difficulty in obtaining a situation is due to his want of decent civilian clothing. A branch of this Society is being started for the purpose of collecting clothing, and the committee will be much obliged if any gentleman having clothing, including shirts and underwear, for which they have no further use, would kindly give it for the use of the men. Arrangements have been made with Messrs. Carter, Paterson and Co., to collect parcels within their area at reduced rates if those giving clothes will kindly send a postcard addressed to the firm at 128 Goswell Road, E.C., requesting them to collect any parcels they may wish to send, or articles of clothing will be received at the Employment Branch of the Soldiers and Sailors Help Society, 115 Ebury Street.

